# Introspective Meditation before Seeking Pleasurable Activities as a Stress Reduction Tool among College Students: A Multi-Theory Model-Based Pilot Study

**DOI:** 10.3390/healthcare10040614

**Published:** 2022-03-25

**Authors:** Manoj Sharma, Amar Kanekar, Kavita Batra, Traci Hayes, Ram Lakhan

**Affiliations:** 1 Department of Social and Behavioral Health, School of Public Health, University of Nevada, Las Vegas, NV 89119, USA; manoj.sharma@unlv.edu; 2 School of Counseling, Human Performance and Rehabilitation, University of Arkansas at Little Rock, Little Rock, AR 72204, USA; axkanekar@ualr.edu; 3 Trauma and Critical Care, Kirk Kerkorian School of Medicine, University of Nevada, Las Vegas, NV 89102, USA; 4 College of Nursing, School of Health Professions, University of Southern Mississippi, Hattiesburg, MS 39406, USA; traci.hayes@usm.edu; 5 Department of Health and Human Performance, Berea College, Berea, KY 40404, USA; lakhanr@berea.edu

**Keywords:** cognitive-behavioral interventions, multi-level model, stress, anxiety, depression, introspective meditation

## Abstract

In the realm of behavioral interventions, a combined approach of yoga and a cognitive-behavioral strategy in the form of introspective meditation (*manan-dhyana*) may offer benefits as a stress management tool. This pilot study focuses on introspective meditation performed before seeking pleasurable activities, which is a self-reflection about whether to pursue a goal that will bring sensory pleasure in life. A non-probability sample of college students was recruited from a mid-sized Southern University of the United States using a 52-items web-based survey built in Qualtrics. Univariate, bivariate, and multivariate statistics were used to analyze data. Of total 65 students, only 21.5% students reported being engaged in the introspective meditation. The sample constituted predominantly females (75.4%), White (64.6%), and undergraduate students (87.7%). The proportions of anxiety, depression, and moderate/high stress were 50.8%, 40.0%, 86.1% respectively. In the hierarchical regression for initiation, the final model explained nearly 21.1% of variance in initiating introspective meditation among participants (*n* = 51) who had not been practicing it. With each unit increment in subscales of initiation (i.e., changes in physical environment), the conditional mean for initiating introspective meditation behavior increased by 0.373 units. In the hierarchical regression for sustenance, the final model explained nearly 50.5% of variance in sustaining introspective meditation behavior among participants (*n* = 51) who had not been practicing it. With each unit increment in subscales of sustenance (i.e., emotional transformation), the conditional mean for sustaining introspective meditation behavior increased by 0.330 units. This study can pave a way for designing interventions for college students to promote introspective meditation directed toward seeking pleasurable activities before engaging in them. This has implications for the reduction of stress as well as a preemptive measure for sexual risk-taking, indulgence in maladaptive behaviors such as smoking, vaping, alcohol, and substance use.

## 1. Introduction

College life is innately stressful, where one can feel overwhelmed while experiencing new social interactions and relationships [1]. According to the recent National College Health Assessment Survey conducted by the American College Health Association (2019) in the United States (U.S.), 87%, 56%, and 45% of college students felt overwhelmed, helpless, and depressed, respectively, which made them less functional in performing their daily and academic activities over the previous year [1,2]. The World Health Organization (WHO) conducted a survey among eight countries with 13,984 college students and found that 35% had a lifetime diagnosis of a mental health disorder [3]. Anxiety disorders, depression, suicide, eating disorders (bulimia, anorexia, and binge eating), bipolar disorder, and schizophrenia were common among college students [1].

A majority of mental health illnesses occur before the mid-twenties and manifest during college years, and are associated with a lower academic functioning [4,5]. A study by Toussaint and colleagues (2016) found that stress degraded physical and mental health in a sample of college students [6]. Stress can be a direct causative factor, an indirect contributory factor, and/or a precipitating factor for mental disorders [7,8,9,10]. Stress among college students is also associated with the adoption of negative coping mechanisms, such as substance use disorders [1]. According to the National Survey on Drug Use and Health (NSDUH) conducted in 2017, approximately 54% of college students consumed alcohol, 34.8% reported being binge drinkers (drank five or more drinks in a single sitting for men or four or more drinks for women), and 9.7% reported being engaged in heavy alcohol use (binged on 5 or more days) over the past month [11]. Reportedly, the annual prevalence of illicit substance use among college students was 45% [12]. Alcohol consumption and illicit drug use were high among college students and point to their poor coping with stressors, although other factors such as peer influence and risk-taking behavior of youth may also be the contributing factors to the increasing prevalence of maladaptive behaviors [13,14]. The immediate gratification of sensory pleasures through alcohol and drugs seems to influence a substantial number of college youth [12,13]. Collective evidence has reported an association between stress-related somatic events and quality of life of students [14]. In addition, one study based on a Swedish sample found an association between stress and early drop-outs from the university [15].

Stressors are generally divided into three types: acute life events (once in a while, discrete, major life influencing events), chronic stressors (that occur on a daily basis), and non-events (when anticipated events do not occur or simple boredom) [10]. A study performed at a university counseling center in Ohio found that the primary stressors among college students include chronic stressors related to academic performance, pressure to succeed, and post-graduation plans [16]. Often students choose activities that provide immediate gratification and sensory pleasures which take away time from academic activities, leading to the stressors [16].

Several modalities of stress management interventions have been implemented among college students. One of the modalities is cognitive-behavioral (CB) stress management. A semester-long study of CB stress management intervention in a group of college students found that it was successful in lowering perceived stress, test anxiety, and personal burnout [17]. Another CB stress management intervention implemented in Korea demonstrated reduction in depression and trait anxiety [18]. A randomized controlled trial (RCT) in Germany using the Internet and App-based CB intervention reduced stress, anxiety, depression, academic work impairment, and increased college-related productivity after seven weeks [19].

Another modality of stress management is yoga, which is a form of mind–body intervention and has been suggested as a means to reduce stress among college students [20]. Yoga is an ancient technique that establishes harmony between mind, body, and the environment [21]. Today, all schools of yoga adhere to the eight classical steps of *ashtanga yoga* promulgated by Patanjali around 200 BC either completely or partially in some form or the other. The eight steps of *Asthanga yoga* are (1) *Yama* (restraint of one’s social conduct for living in harmony within society), (2) *Niyama* (observances or rules for self), (3) *Asana* (low-impact physical activity postures), (4) *Pranayama* (breathing regulation), (5) *Pratyahara* (withdrawal of senses from their objects), (6) *Dharana* (concentration of the mind at certain points), (7) *Dhyana* (meditation), and (8) *Samadhi* (union with the primordial state) [21]. A combined approach of the components of yoga with a cognitive-behavioral approach in the form of introspective meditation (*manan-dhyana*) or reflective thinking on one’s own thinking has been proposed as a means to reduce unwanted stress [22]. Some introspective meditations are on seeking pleasure, achieving security, circumventing anger, escaping jealousy, etc. This study focuses on introspective meditation (*manan-dhyana*) performed before seeking pleasurable activities, which is a self-reflection about whether to pursue a goal that will bring sensory pleasure in life. Sensory pleasure for this study is defined as any gratification obtained through the enjoyment of the five senses (touch, sight, smell, hearing or taste) with an external object. This has implications for the reduction of stress as well as a preemptive measure for sexual risk-taking, and indulgence in maladaptive behaviors such as smoking, vaping, alcohol, and substance use. While *manan-dhyana* has not been studied explicitly in the past, previous literature has explored the potential of a similar concept of problem-solving skills in addressing a myriad of maladaptive behaviors such as alcohol use [23], aggression [24], and general stress [25].

In the realm of theory, a fourth-generation theoretical paradigm of multi-theory model (MTM) of health behavior change [26,27] was used in this study to ascertain factors among college students that will foster initiation and maintenance of behavior of introspective meditation every day for 20 min on the pleasure-seeking activities they plan to undertake that day. MTM has been used with a variety of health behaviors among college students, such as physical activity [28], small portion size consumption for reducing calorie intake to prevent obesity [29], and adequate sleep [30]. MTM proposes htat the constructs of *participatory dialogue* (in which the advantages of a health behavior change outweigh the disadvantages), *behavioral confidence* (which is the futuristic confidence in one’s ability to make the behavior change), and *changes in the physical environment* (which is the availability and accessibility of tangible resources) are essential to starting a health behavior change, which in this case was daily introspective meditation for 20 min [31]. MTM further proposes that for maintaining the behavior change, the constructs of *emotional transformation* (or converting negative emotions into goals), *practice for change* (or constant thinking and rethinking about the behavior change) and *changes in the social environment* (or the social support) are vital. It is against this backdrop that the aim of this study was to use MTM to explain the initiation and maintenance of the behavior of introspective meditation about seeking pleasure as a tool for the reduction of stress among college students.

## 2. Materials and Methods

### 2.1. Study Design and Sample

This cross-sectional pilot study was conducted at a mid-sized Southern University of the U.S. with over 9000 students. Potential participants were invited via the research system housed in the Psychology Department at the University, via an e-mail announcement of the study on the classifieds listserv, and by approaching the faculty teaching online courses to post an e-mail announcement in their online courses. The non-probability (quota) sampling was used to recruit the sample. The participants comprised all full time and part-time college students attending the University who were above the age of 18 years. Only students who understood the English language and possessed the ability to provide informed consent were included.

### 2.2. Ethical Considerations

The study was approved by the University Institutional Review Board (IRB) (Protocol # 19–152–M1). Students were invited to fill out a web-based survey using the Qualtrics platform. No identifying names and contact information of the participants were solicited, making it an anonymous study. No compensation or incentive was provided to the participants for participation in the study. Participation in the survey was completely voluntary, and the participant could choose to leave the survey at any point in time.

### 2.3. Survey Instrument

The study used a combination of psychometric valid (e.g., Perceived Stress Scale and Patient Health Questionnaire) and self-designed instruments (based on MTM) consisting of a total of 52 items. The MTM tool was validated for face and content validity by a panel of six experts in two iterations. The experts were those familiar with instrumentation in behavioral sciences (*n* = 6), with introspective meditation (*n* = 6), MTM (*n* = 5), and college students (*n* = 4). The experts were provided with the instrument and operational definitions of all constructs. Between the first round and second round, the readability of the instrument was improved based on the feedback received and the definition of sensory pleasure was added to the directions. The Flesch-Kincaid reading level of the final instrument was 7.3 (or less than eight grade), and Flesch-Kincaid reading ease was 55.8. In addition, psychometrically valid tools such as Perceived Stress Scale [32] and PHQ–4 [33] were also used.

**Figure 1:**
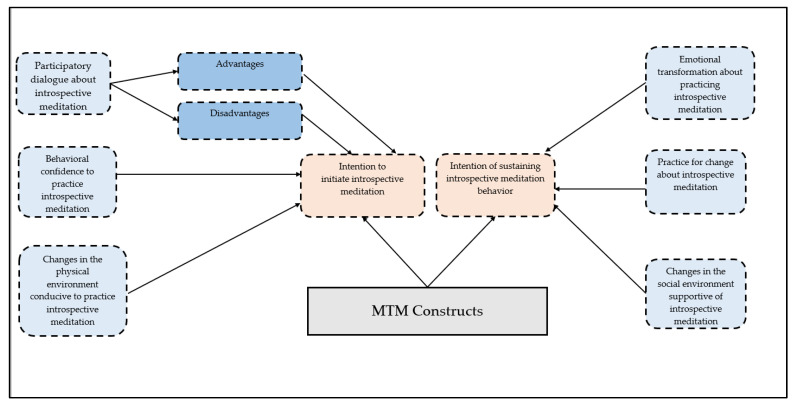
Multi-theory model framework.

To summarize the instrument, questions 1–9 were demographic questions; questions 10–19 pertained to perceived stress on a rating scale of never (0) to very often (4), with a possible range of 0–40 units; questions 20–23 were PHQ–4 with total scores ranging from 0 to 12 units, with categories of psychological distress being: none (0–2 units), mild (3–5 units), moderate (6–8 units), severe (9–12 units). Questions 24–28 were about the construct of advantages on a rating scale of never (0) to very often (4); and questions 29–33 were about the construct of disadvantages on a rating scale of never (0) to very often (4). The score of the participatory dialogue construct was obtained from subtracting the summative score of disadvantages from the summative score of advantages and ranged from −20 to +20 units. Questions 34–38 tapped into the construct of behavioral confidence on a rating scale of not at all sure (0) to completely sure (4) and a summative score yielded a possible range of 0–20 units. Likewise, summative scores for changes in the physical environment from questions 39–41; emotional transformation from questions 42–44; practice for change from questions 45–47; and changes in the social environment from questions 48–50 using a rating scale of not at all sure (0) to completely sure (4) yielded total scores of 0–12 units. Question 51 pertained to the intention to start the practice of introspective meditation for seeking pleasure for 20 min daily in the upcoming week rated on a scale of not at all sure (0) to completely sure (4). Final question 52 was about continuing the practice of introspective meditation for seeking pleasure for 20 min daily from now on rated on a scale of not at sure (0) to completely sure (4). All MTM constructs are indicated in Figure 1.

### 2.4. Models

Two models were built in this study. For Model 1, initiation, which is defined as the intention to start or initiate the practice of introspective meditation for seeking pleasure for 20 min/daily in the “upcoming week”, was used as the dependent variable, while the constructs of participatory dialogue, behavioral confidence, and changes in the physical environment were used as independent variables. For Model 2, sustenance, which is defined as the intention to continue the practice of introspective meditation for seeking pleasure for 20 min/daily from now onwards, was used as the dependent variable. The constructs of emotional transformation, practice for change, and changes in the social environment were used as independent variables. The perceived stress level and the level of psychological distress were also assessed in this study.

### 2.5. Sample Justification

Given the pilot nature of this study, we used the flat sample size rule of sample size recommendations provided by previous simulation and trial studies [34,35,36]. The recommended pilot sample size ranged from 30 to 70 subjects, which aligned well with the sample used in this study [31,32,33]. For regression, the *p*-value was set at 0.05, power at 0.80, the effect size was considered at 0.35 (as is the case in social and behavioral science research) [37,38], and there were 8 predictors in each model. These assumptions yielded a sample size of 52 which was inflated by 10% for any missing values to arrive at the sample size of 57 participants, which aligned well with the sample used (*N* = 65) in this study.

### 2.6. Data Analysis

Survey responses were imported to IBM SPSS version 27.0 (IBM Corp., Armonk, NY, USA) for the analyses. For assessing the construct validity of the MTM tool, Confirmatory factor analysis (CFA) with a maximum likelihood method was used. Reliability diagnostics were assessed to check the internal consistency of the MTM instrument. For establishing a one-factor solution following the Kaiser criterion of Eigenvalue greater than or equal to 1.0, factor loadings on each item greater than 0.610 (after doubling the critical value for a sample size of 65 at an α = 0.01 for a two-tailed test) were established as a priori, given the generally accepted recommendations from previous studies [36,37]. Categorical variables were represented as frequencies and proportions, whereas continuous variables were represented by mean and standard deviations. Anxiety, depression, and stress were coded per scoring criteria indicated by previous studies [32,33]. The normal approximation to the binomial distribution method was used to calculate 95% confidence intervals of proportions in the univariate analyses. A bivariate Pearson’s correlation test was performed to calculate correlations among the MTM variables. Two models of hierarchical multiple regression (HRM) were fit to predict the likelihood of initiation and sustenance of introspective meditation among students who were not currently practicing it. All assumptions of HRM, including independence of observations, linearity, homoscedasticity, multicollinearity, and normality, were assessed. A detailed strategy of HRM model building can be seen in Table 1. All *p*-values are two-sided.

## 3. Results

### 3.1. Demographics and Psychological Profile

A total of 65 valid responses were analyzed, of which only 21.5% of students reported being engaged in the introspective meditation. The sample constituted predominantly females (75.4%), White (64.6%), and undergraduate students (87.7%, Table 2). The mean age of the sample was 27.72 ± 11.9 years. Nearly 3/4th of the sample reported being employed and over 65% participants had income under $50,000 (Table 2). The proportions of anxiety, depression, and moderate/high stress were 50.8%, 40.0%, 86.1%, respectively (Table 2). Among students with depression (*n* = 26), nearly 33% had mild depression and only 8% had moderate depression. Of 33 students who reported having anxiety, 19 (29.2%) had mild anxiety and 14 (21.5%) had moderate anxiety.

### 3.2. Perceived Stress

**Figure 2:**
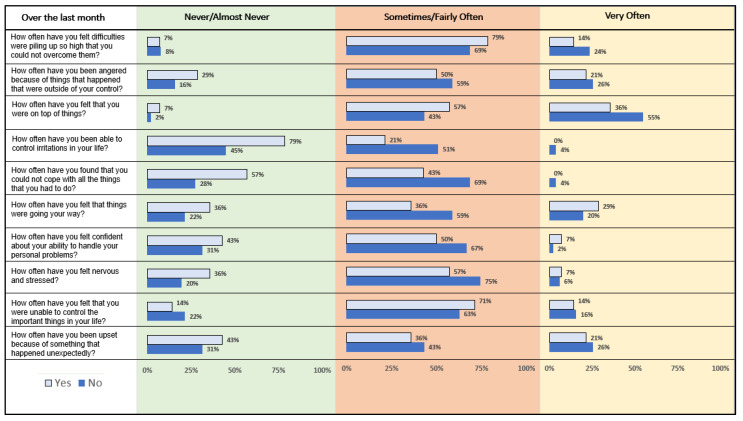
Item-wise analysis of perceived stress reported by college students engaged or not engaged in introspective meditation. Figure legend: Yes, corresponds to students engaged in the introspective meditation; No, corresponds to the students who were not engaged in the introspective meditation.

Figure 2 displays item-wise proportion of perceived stress reported by the respondents. Seventy-nine percent of students who were engaged in the introspective meditation reported that they have never/almost never been able to control irritations in their life over the past month compared to 45% students who were not engaged in the introspective meditation. These differences were statistically non-significant. Fifty-five percent of students who were not engaged in the introspective meditation felt (very often) they were on the top of the things as compared to 36% of students who were engaged in the introspective meditation, although these results were statistically non-significant (Figure 2).

### 3.3. Bivariate Statistics

As indicated in Table 3, perceived advantages were directly correlated with behavioral confidence (r = 0.35, *p* < 0.01). Perceived disadvantages were indirectly correlated with changes in the physical environment (r = −0.45, *p* < 0.01), and emotional transformation (r = −0.25, *p* < 0.05). Behavioral confidence was directly correlated with changes in the physical environment (r = 0.37, *p* < 0.01), emotional transformation (r = 0.56, *p* < 0.01), practice for change (r = 0.38, *p* < 0.01), and changes in the social environment (r = 0.28, *p* < 0.05). Changes in physical environment were directly correlated with the emotional transformation (r = 0.64, *p* < 0.01), practice for change (r = 0.49, *p* < 0.01), and changes in social environment (r = 0.35, *p* < 0.01). A strong and direct correlation was observed between emotional transformation and practice for change (r = 0.75, *p* < 0.01). Reliability diagnostic test indicated that the Cronbach’s Alpha of the entire MTM scale was 0.85, which was deemed appropriate given the previous literature [35]. Construct validation yielded a one-factor solution for each MTM subscale and satisfied Eigen’s criteria. All factor loadings were above the critical value of 0.610, as specified earlier in the Materials and Methods section.

### 3.4. Hierarchical Regression

In the hierarchical regression for initiation, the final model (Model 4) explained nearly 21.1% of variance in initiating introspective meditation behavior among participants (*n* = 51) who had not been practicing introspective meditation (R^2^ = 0.346, F (8, 39) = 2.575, *p* < 0.05; adjusted R^2^ = 0.211, Table 4). With each unit increment in subscales of initiation (i.e., changes in physical environment), the conditional mean for initiating introspective meditation behavior increased by 0.373 units (Model 4, Table 4). None of the slopes of demographic and other MTM variables were significant, which indicated no significant differences in the conditional mean changes in initiating introspective meditation among participants who had not been practicing it. In the hierarchical regression for sustenance, the final model (Model 4) explained nearly 50.5% of variance in sustaining introspective meditation behavior among participants (*n* = 51) who had not been practicing it (R^2^ = 0.589, F (8, 39) = 6.988, *p* < 0.001; adjusted R^2^ = 0.505, Table 5). With each unit increment in subscales of sustenance (i.e., emotional transformation), the conditional mean for sustaining introspective meditation behavior increased by 0.330 units (Model 4, Table 5). None of the slopes of demographic or other MTM variables were significant, which indicated no significant differences in the conditional mean changes in sustaining introspective meditation among participants who had not been practicing it.

## 4. Discussion

The purpose of this study was to focus on the practice of introspective meditation (*manan-dhyana*) before engaging in sensory pleasures in life. The multi-theory model of health behavior change was used as the underlying theoretical framework. Although it is a newer and more recent theory, constructs of this theory have already been used extensively as a predictive model for a variety of health behaviors [27]. To our knowledge, the application of the MTM theory in assessing introspective meditation behavior for sensory pleasure seeking among college students is the first in predicting reflective thinking in this group. However, previous study tested the MTM model in predicting the intent to practice meditation among the general U.S. population [21,27].

In the present study, the construct of ‘changes in the physical environment’ was found to be a significant predictor of likelihood of initiation of introspective meditation before seeking pleasurable activities among college students, who were not engaged in the practice. Changes in the physical environment were found to be important, as it was clearly shown that physical environments, such as availability of space for meditation, accessibility, and resources for initiating the practice, are lacking among college students, who have a busy academic schedule, work responsibilities, and additional family commitments. This construct could be modified by providing classes on this technique, involving students in various peer-to-peer networks, linking with resources on campus community such as counseling and the student support services.

Another construct of MTM, behavioral confidence, showed significance only in Model 3 of the hierarchical regression. This significance may be considered cautiously in driving behavior change towards initiation of introspective meditation through behavioral confidence. Since it alludes to the future behavior of the participants, it could be considered that college students may have a predilection to modify their behaviors at a time in future related to sensory pleasure seeking then in the present circumstances. Furthermore, in the absence of an intervention, many students would not have been perceptive about their confidence levels regarding this construct, leading to it being statistically non-significant. Participatory dialogue, which is two-way communication emphasizing the advantages of introspective meditation facilitated by faculty, counselors, peer-to-peer or health professionals for students was not found to be a significant predictor in the likelihood of initiating introspective meditation in this study. While this is a robust construct based on several value expectancy theories, like the transtheoretical model, social cognitive theory, health belief model, etc. [27], it seemed to have limited application for this behavior and for the college students as a group. This could be due to the lack of an intervention and the students’ preconceived notion about the simplicity of the technique with respect to its making a significant difference in their life. Future interventions should have activities that facilitators can use to emphasize the potential benefits of *manan-dhyana* before engaging in sensory pursuits for the college students [22].

Looking at the sustenance model of the MTM, the construct of ‘emotional transformation’ was found to be a significant predictor of likelihood for sustenance of introspective meditation (*manan-dhyana*) before seeking sensory pursuits among college participants who were not engaged in the sustained practice of introspective meditation. It accounted for about 50% of the variance in explaining the intent to continue the practice of *manan-dhyana*, which is quite promising for future intervention work. The modification of emotional transformation entails creating an environment for college students which self-motivates them to transform their negative feelings into positive goals. It also requires overcoming self-doubt and additional personal emotional challenges in order to accomplish goals [39,40]. This could be again modified by making use of campus resources such as counseling resources. Additional aspects that could be addressed include self-awareness, empathy building, and managing relationships as part of the emotional intelligence theory [22,28]. Some of the components of emotional intelligence which explain the mental health component are well-being, self-control, and sociability [41,42,43]. The construct of emotional transformation in the context of *manan-dhyana* can be influenced by enhancing self-identification of emotions and channeling those into specific self-reflective goals to be implemented before engaging in any sensory pursuit.

Furthermore, the construct of practice for change was not significant in our study, though this is an important construct of MTM. This could be due to the fact that practice for change entails reflective thinking by the students and making conscious attempts at practicing the behavior, and introspective meditation is that in its own right. This may have interfered with the interpretability of this construct by the respondents [21,22]. However, for interventions, it would be beneficial if students can be asked to maintain a digital diary or a journal where they can record their reflections on engaging in the introspective meditation activity. Finally, the construct of changes in the social environment was not found to be significant in this study. This could be due to the personal nature of *manan-dhyana*, and many students may have been reluctant to share about doing this activity or soliciting support from others in performing this activity. 

### 4.1. Strengths and Limitations

To our knowledge, this is the first pilot study to use a theoretical paradigm to study the practice of *manan-dhyana* before seeking sensory pursuits by college students. This study could pave way for the design of interventions for college students to promote introspective meditation directed toward seeking pleasurable activities before engaging in them. This would have implications not only for stress management, but also promote safer sex behaviors, resistance to vaping, smoking, drugs, alcohol use and other maladaptive behaviors.

Although this study used a newer health behavior change model and tested it for a behavior among college students, it has some limitations. First, the sample size of this study with college students was very small, and the sample was sourced from a single institution. Hence, there is limited generalizability of the results. Future studies should collect data from multiple institutions with larger sample sizes. Second, the campus racial diversity of the college students could not be captured well due to the majority of participants in the study being White. Hence, the results of this study may not be transferable to other campuses that have broader racial diversity. In addition, some unmeasured variables, such as substance abuse, alcohol consumption, smoking, and major of the students may have introduced some residual confounding. Third, this was a cross-sectional study (snapshot) study at a specific point in the academic year. One of the limitations of using a cross-sectional study design is that a temporal causal relationship between the study variables cannot be determined and the relationships are limited to correlations and associations. Fourth, since this was an online survey, it is assumed that the participants completing the survey were truly the participants involved in the study. Additionally, in survey studies using self-report, there is always an element of ‘social desirability bias that happens when respondents mark responses which portray a favorable image of themselves [41].

### 4.2. Implications for Practice and Conclusions

Stress is a pervasive factor among the lives of college students. As mentioned earlier in this article, various stress reduction techniques have been tried to alleviate stressors in the lives of college students such as cognitive behavioral therapy [43], meditation [44,45], and yoga [20,46]. Introspective meditation, or ‘*Manan-Dhyana*’, purports to create a reflective ability among college students before seeking sensory pleasures and mitigation of stress. This study presents a robust framework of the multi-theory model of health behavior change in predicting this behavior change among a sample of college students at a southern university. In terms of initiation of this behavior, we need to be purposeful in considering environmental factors such as availability and accessibility of venues to initiate this behavior on college campuses and pay a sustained attention to emotional transformation among students. 

Environmental factor modification can be achieved by interventions devised at the ecological level which involves not just the students, but administration, staff, and student support services to play a joint role in creating spaces, avenues, and support services to make a favorable environment conducive to health behavior change. In the case of *manan-dhyana* among college students, colleges and universities should organize classes, provide space for meditation on campuses, and channel counseling and other services to support such practices. Similarly, for long-term behavior change and sustenance, the tenets of emotional intelligence need to be infused in services offered at college campuses across the nation. These could be either integrated through counseling interventions or behavior change interventions, which can also be offered through social media to capture the youth population [47]. 

Finally, future studies need to be devised using a larger sample size along with increasing the diversity of the student population across various campuses in the nation to test this theory with a robust design involving all the constructs of the theory. These attempts at modification of health behaviors for an improved mental status are the need of the hour, particularly amid a pandemic. In addition, assessing the association between practicing introspective meditation and other maladaptive behaviors can be the focus for future studies. 

## Figures and Tables

**Table 1:** Hierarchical multiple regression model building algorithm.

**Initiation Model**	
Model 1	Initiation = Intercept + Age + Gender + Race + Employment + Type of student
Model 2	Initiation = Intercept + Model 1 variables + Participatory dialogue
Model 3	Initiation = Intercept + Model 2 variables + Behavioral confidence
Model 4	Initiation = Intercept + Model 3 variables + Changes in the physical environment
**Sustenance Model**	
Model 1	Sustenance = Intercept + Age + Gender+ Race + Employment + Type of student
Model 2	Sustenance = Intercept + Model 1 variables + Emotional transformation
Model 3	Sustenance = Intercept + Model 2 variables + practice for change
Model 4	Sustenance = Intercept + Model 3 variables + changes in the social environment

**Table 2:** Univariate demographic statistics and psychological profile of the study population (*N* = 65).

Variable	Categories	*n* (%)	95% CI (LCL, UCL)
Engaged in Introspective Meditation	Yes	14 (21.5)	12.3, 33.5
	No	51 (78.5)	66.5, 87.7
Gender	Male	14 (21.5)	12.3, 33.5
	Female	49 (75.4)	63.1, 85.2
	Other	2 (3.0)	0.4, 10.7
Age in years (Mean ± SD)	-	27.72 ± 11.9	24.77, 30.7
Race/ethnicity	White	42 (64.6)	51.7, 76.1
	Non-White	23 (35.4)	23.9, 48.2
Current Education Status	Undergraduate	57 (87.7)	77.2, 94.5
	Graduate	8 (12.3)	5.5, 22.8
Employed	Yes	49 (75.4)	63.1, 85.2
	No	16 (24.6)	14.7, 36.8
Income	Less than $50,000	41 (63.1)	50.2, 74.7
	$50,000–$100,000	14 (21.5)	12.3, 33.5
	$100,000–$150,000	2 (3.1)	0.4, 10.7
Anxiety	Yes	33 (50.8)	38.1, 63.4
	No	32 (49.2)	36.6, 61.9
Depression	Yes	26 (40.0)	28.0, 52.9
	No	39 (60.0)	47.1, 71.9
Stress	Low	9 (13.8)	6.5, 24.6
	Moderate	32 (49.2)	36.6, 61.9
	High	24 (36.9)	25.3, 49.8

Note: Some percentages may not add up to 100%, as a few participants preferred not to answer; CL: Confidence intervals; LCL: Lower Confidence Level; UCL: Upper Confidence Level.

**Table 3:** Bivariate correlations and reliability diagnostics for MTM variables (*N* = 65).

Variables	1	2	3	4	5	6	7
1. Perceived Advantages	-	−0.23	0.35 **	0.04	0.19	0.19	0.14
2. Perceived Disadvantages	−0.23	-	−0.23	−0.45 **	−0.25 *	−0.19	−0.08
3. Behavioral Confidence	0.35 **	−0.23	-	0.37 **	0.56 **	0.38 **	0.28 *
4. Changes in the Physical Environment	0.04	0.45 **	0.37 **	-	0.64 **	0.49 **	0.35 **
5. Emotional Transformation	0.19	−0.25 *	0.56 **	0.64 **	-	0.75 **	0.43 **
6. Practice for Change	0.19	−0.19	0.38 **	0.49 **	0.75 **	-	0.60 **
7. Changes in the Social Environment	0.14	−0.08	0.28 *	0.35 **	0.43 **	0.60 **	-

* Significant below 0.05; ** Significant below 0.01.

**Table 4:** Hierarchical regression to predict likelihood for initiation of introspective meditation among participants who were not engaged in the practice (*n* = 51).

Variables	Model 1		Model 2		Model 3		Model 4	
	B	*β*	B	*β*	B	*β*	B	*β*
Initiation as a dependent variable								
Constant	2.345	*-*	2.333	*-*	0.983	*-*	0.500	*-*
Age	−0.008	−0.082	−0.008	−0.078	−0.001	−0.003	−0.007	−0.070
Gender (Ref: Female)	−0.386	−0.135	−0.375	−0.132	−0.401	−0.141	−0.351	−0.123
Race (Ref: White)	−0.102	−0.041	0.034	0.014	0.139	0.056	0.0007	0.001
Employment status (Ref: No)	−0.525	−0.178	−0.687	−0.233	−0.586	−0.198	−0.302	−0.102
Type of student (Ref: Undergraduate)	0.609	0.168	0.431	0.119	0.337	0.093	0.247	0.068
Participatory dialogue	-	-	0.051	0.216	0.018	0.078	0.001	−0.001
Behavioral confidence	-	-	-	-	0.128	0.390 *	0.077	0.233
Changes in the physical environment	-	-	-	-	-	-	0.184	0.373 *
R^2^	0.091	-	0.130	-	0.254	-	0.346	-
F	0.839	-	1.021	-	1.945	-	2.575 *	-
Δ R^2^	0.091		0.039		0.124		0.092	
Δ F	0.839	-	1.846	-	6.648*	-	5.462 *	-

* *p*-value < 0.05; Adjusted R^2^ initiation = 0.211.

**Table 5:** Hierarchical regression to predict likelihood for sustenance of introspective meditation among participants who were not engaged in the practice (*n* = 51).

Variables	Model 1		Model 2		Model 3		Model 4	
	B	*β*	B	*β*	B	*β*	B	*β*
Sustenance as a dependent variable								
Constant	1.449	*-*	−0.999	*-*	−0.948	*-*	−1.017	
Age	−0.004	−0.039	0.009	0.086	0.006	0.060	0.005	0.045
Gender (Ref: Female)	−0.027	−0.009	0.065	0.022	0.001	0.001	−0.038	−0.013
Race (Ref: White)	−0.0260	−0.102	−0.0378	−0.0148	−0.386	−0.151	−0.338	−0.132
Employment status (Ref: No)	0.041	0.013	0.525	0.172	0.430	0.141	0.410	0.135
Type of student (Ref: Undergraduate)	0.890	0.238	0.400	0.107	0.511	0.137	0.681	0.182
Emotional transformation	-	-	0.320	0.665 **	0.172	0.357 *	0.159	0.330 *
Practice for change	-	-	-	-	0.208	0.426 *	0.146	0.299
Changes in the social environment	-	-	-	-	-	-	0.097	0.233
R^2^	0.069	-	0.463	-	0.557	-	0.589	-
F	0.619	-	5.893 **	-	7.196 **	-	6.988 **	-
Δ R^2^	0.069	-	0.394	-	0.094	-	0.032	-
Δ F	0.619	-	30.113 **	-	8.527 *	-	3.004	-

* *p*-value < 0.05; ** *p*-value < 0.001; Adjusted R^2^ sustenance = 0.505.

## Data Availability

The data presented in this study are available on request from the corresponding author. The data are not publicly available due to ethical reasons.
